# Role of extracellular vesicle-associated proteins in the progression, diagnosis, and treatment of hepatocellular carcinoma

**DOI:** 10.1186/s13578-024-01294-6

**Published:** 2024-09-03

**Authors:** Yao-Ge Liu, Shi-Tao Jiang, Jun-Wei Zhang, Han Zheng, Lei Zhang, Hai-Tao Zhao, Xin-Ting Sang, Yi-Yao Xu, Xin Lu

**Affiliations:** Department of Liver Surgery, Peking Union Medical College Hospital, Chinese Academy of Medical Sciences & Peking Union Medical College (CAMS & PUMC), Beijing, China

**Keywords:** Hepatocellular carcinoma (HCC), Extracellular vesicles (EVs), EV-associated proteins, Tumor microenvironment (TME), Proteomics, Biomarkers

## Abstract

Hepatocellular carcinoma (HCC) is the most common type of primary liver cancer, characterized by difficulties in early diagnosis, prone to distant metastasis, and high recurrence rates following surgery. Extracellular vesicles (EVs) are a class of cell-derived particles, including exosomes, characterized by a phospholipid bilayer. They serve as effective carriers for intercellular communication cargo, including proteins and nucleic acids, and are widely involved in tumor progression. They are being explored as potential tumor biomarkers and novel therapeutic avenues. We provide a brief overview of the biogenesis and characteristics of EVs to better understand their classification standards. The focus of this review is on the research progress of EV-associated proteins in the field of HCC. EV-associated proteins are involved in tumor growth and regulation in HCC, participate in intercellular communication within the tumor microenvironment (TME), and are implicated in events including angiogenesis and epithelial-mesenchymal transition (EMT) during tumor metastasis. In addition, EV-associated proteins show promising diagnostic efficacy for HCC. For the treatment of HCC, they also demonstrate significant potential including enhancing the efficacy of tumor vaccines, and as targeting cargo anchors. Facing current challenges, we propose the future directions of research in this field. Above all, research on EV-associated proteins offers the potential to enhance our comprehension of HCC and offer novel insights for developing new treatment strategies.

## Introduction

Primary liver cancer ranked as the sixth most frequently diagnosed cancer and was the third highest cause of cancer-related mortality worldwide in 2020 [1]. Hepatocellular carcinoma (HCC), as the most common form (comprising 75-85% of cases) of primary liver cancer, is characterized by insidious onset and a complex progression of biological processes. Due to the delayed manifestation of symptoms, HCC is diagnosed at advanced stages in over 50% of patients, which rendered the only potentially curative surgical approach unfeasible [2]. Despite achievements in the research of HCC, challenges including tumor regulation, early diagnosis strategies, and personalized precision medicine approaches still persist. Facing these challenges, extracellular vesicles (EVs) are extensively researched as promising hotspots based on their biological characteristics and cancer-specific cargo.

EVs are defined as particles surrounded by a lipid bilayer membrane that are discharged by various cells and cannot replicate independently [3]. According to the distinctions in size and specific origins of EVs, the nomenclature of EVs often encompasses various types. For example, small EVs are often described as < 200 nm in diameter while large EVs are often > 200 nm. Another common nomenclature, “exosome”, is a biogenesis-related term that specifically refers to vesicles originating from the endosomal system. Exosomes represent a subtype of small EVs with an intraluminal diameter < 200 nm. On account of the typically challenging experimental verification of the subcellular origin of exosomes, broad claims regarding research on exosomes may not be entirely accurate [4–7]. Currently, there are no recognized molecular markers of EVs or EV subtypes. The most widely tested tetraspanins CD9, CD63, and CD81 are often used to characterize exosomes, but they are not specific markers for exosomes [8]. As a consensus, the Minimal information for studies of extracellular vesicles (MISEV 2023) recommended extracellular vesicles as a standard nomenclature. Therefore, unless otherwise specified in the study, we strive to use “EVs” as a standard term in this review to summarize the relevant research as comprehensively as possible.

The tumor microenvironment (TME) plays a substantial role in the development of HCC, while HCC often presents an immunosuppressive TME. Stromal cells represented by macrophages and cancer-associated fibroblasts participate in the process of limiting immune cell infiltration [9], subsequently hindering the efficacy of immunotherapy. EVs serve as effective carriers containing a variety of substances including nucleic acids (miRNA, long noncoding RNA, and DNA), proteins, lipids, and glycoproteins [10], which provide a convenient approach for intercellular communication, and crosstalk between tumors and TME. The distinctive vesicular structures and targeted membrane signaling molecules of EVs enabled wide involvement in intercellular substance transfer and information exchange. Therefore, the properties of EVs have garnered great attention for exploring their role in biological functions, tumorigenesis, metastasis, angiogenesis, and drug resistance of HCC [11].

Moreover, EVs have exhibited promising capabilities in both the diagnosis and treatment of HCC. Alpha-fetoprotein (AFP), predominantly secreted by HCC cells, is recognized as a significant tumor biomarker for diagnosing HCC. Nonetheless, its sensitivity varies between 25% and 65% [12]. The exploration of novel biomarkers of HCC has never ceased. EVs have emerged as promising targets for HCC early diagnosis primarily based on their accessibility and specificity. Firstly, EVs are widely present in bodily fluids, including blood, urine, saliva, etc. [13]. This facilitates easy retrieval of EVs. On the other hand, the formation of EVs involves the process of inward budding from membrane and materials exchange with the endoplasmic reticulum and Golgi apparatus [14]. Consequently, EVs carry membrane and cytosolic proteins from the originating cells that accurately reflect the molecular features of the original tumor. These properties offer the potential for minimal tumor early diagnosis by using EVs as biomarkers. Furthermore, EVs as endogenous delivery carriers have the advantage of reduced immunogenicity, heightened biocompatibility, and increased permeability through cell membranes [15], they can serve as excellent drug delivery medium after undergoing bioengineering modifications. In terms of transport reliability, the protective biological membrane of EVs can guard their payload against degradation. By utilizing EVs as drug carriers, it is possible to enhance innate and tumor-specific immune responses [16, 17], promote apoptosis of HCC [18], and bolster the activity of targeted therapy [19].

In this review, we focused on the proteins from EVs in the field of HCC. As EV membranes contain a rich variety of protein components, as well as cargo with diverse protein constituents, proteomics studies on EVs can help with understanding the roles that proteins play in biological processes, including cell signaling, metabolism, and disease. By reviewing current research, we aim to gain insights into the tumor progression and TME of HCC from the perspective of EVs, summarize potential protein biomarkers and new therapeutic approaches, and furthermore discuss potential research directions in this field.

## Biogenesis and characteristics of EVs

Currently, the classification of EV subtypes is challenged by experimental detection and isolation methods, where EVs mainly refer to two main categories, exosomes and ectosomes [20]. Ectosomes are vesicles secreted directly from the plasma membrane by direct outward budding, with a larger diameter ranging from 50 to 1000 nm compared to exosomes [21]. Although research on ectosomes of HCC is scarce, there are still studies demonstrating the role of ectosomal proteins such as PKM2 in reshaping the tumor microenvironment and its potential role as a biomarker of HCC [22]. In comparison, exosomes have attracted more attention in the research field due to their formation through a unique intracellular regulatory mechanism, which makes the exploration of their compositions and functions more appealing. Current research predominantly focuses on small EVs, often referred to as “exosomes.” Therefore, for a better understanding of the characteristics of exosomes, it is essential to provide a detailed explanation of their biogenesis process.

**Fig. 1:**
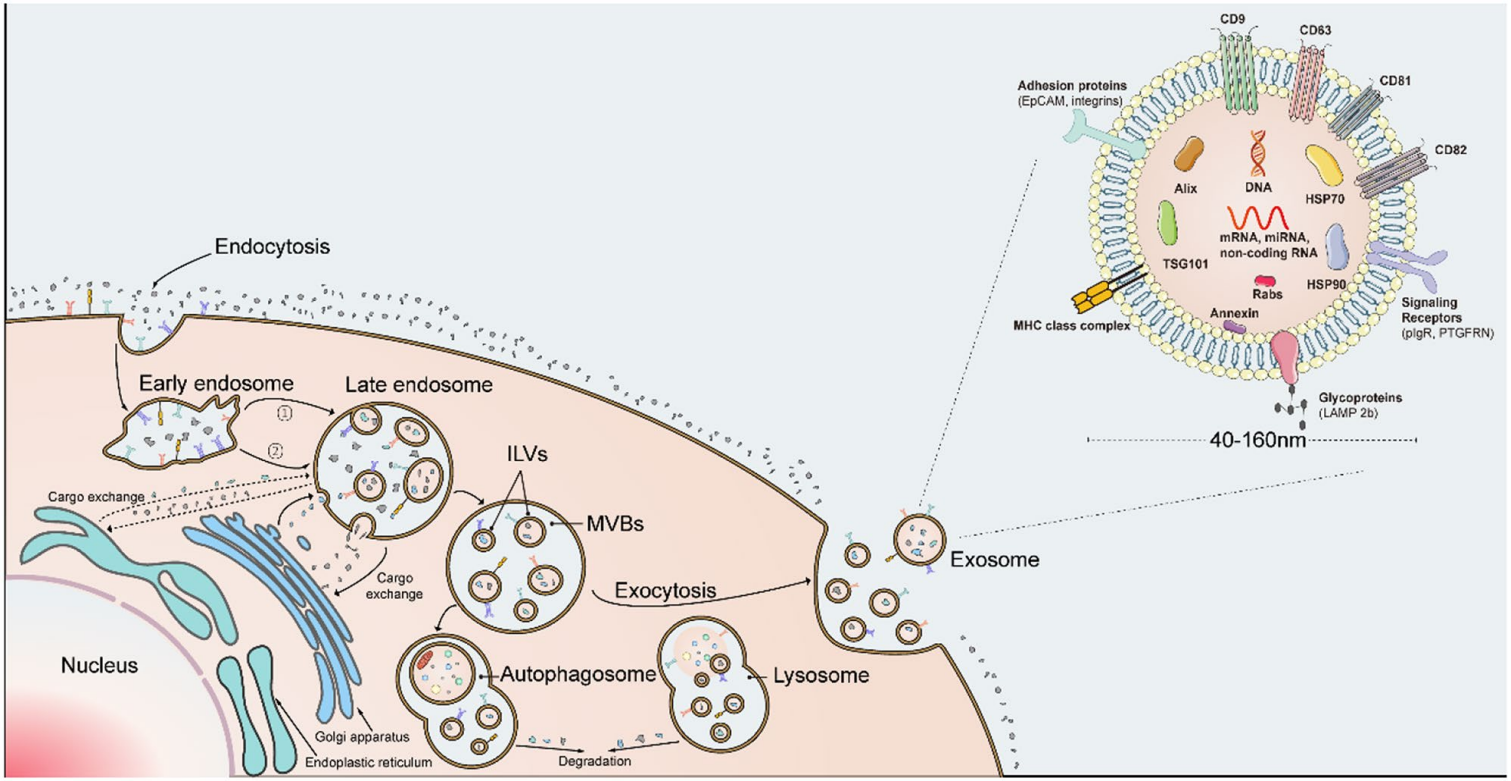
The secretion process and structure of exomes. Endosomes are generated through cellular invagination and further invaginate to exchange components with the intracellular membrane system, forming intraluminal vesicles (ILVs), which further develop into multivesicular bodies (MVBs). Endosomes can either be degraded within the cell or release ILVs through exocytosis to become exosomes. Exosomes have a lipid bilayer structure, with several characteristic membrane and cargo components labeled in the figure. 1. ESCRT-dependent pathway; 2. ESCRT-independent pathway

The generation of exosomes initially begins with inward budding from the plasma membrane, and in this process, endosomes are generated. During the maturation of endosomes, they undergo material exchange with the endoplasmic reticulum and the Golgi apparatus [14]. The endosomal membrane invagination of mature endosomes forms multiple intraluminal vesicles (ILVs), as precursors of exosomes, which further develop into multivesicular bodies (MVBs). Ultimately, ILVs can be discharged as exosomes through exocytosis, after the membrane of MVBs merges with the plasma membrane, or are degraded by lysosomes or autophagosomes (Fig. 1). In summary, exosome formation involves two steps of membrane invagination, resulting in the structure of large vesicles known as MVBs containing smaller ILVs. These smaller vesicles, upon secretion from the cell, are referred to as exosomes, typically with diameters ranging from 40 to 160 nm [21].

The sorting machinery of ILVs can rely on both the ESCRT (Endosomal sorting complexes required for transport)-dependent pathway and the ESCRT-independent pathway. The proposal of ESCRT were based on studies of a series of vacuolar protein sorting (VPS) mutants in budding yeast [23–25]. The endosomal sorting complexes are a kind of cytosolic protein complexes, which are currently recognized as four subtypes: ESCRT-0, ESCRT-I, ESCRT-II, and ESCRT-III. The endosomal sorting complexes, together with other accessory proteins including Vps4 and Bro1, participate in the process of endosomal sorting of ubiquitinated cargo proteins [26–28]. Typically, the ubiquitylation of proteins in the form of lysine-63-linked polyubiquitin chains functions as sorting signals for endocytosis [29]. The ubiquitinated proteins are sorted into ILVs through the sequential action of the ESCRT complexes, while researchers noticed that ESCRT-0, -I, and -II all possess subunits that could bind to ubiquitin and interact directly with ubiquitylated cargos, which provided supporting evidence for the ESCRT-independent pathway [24, 25].

**Fig. 2:**
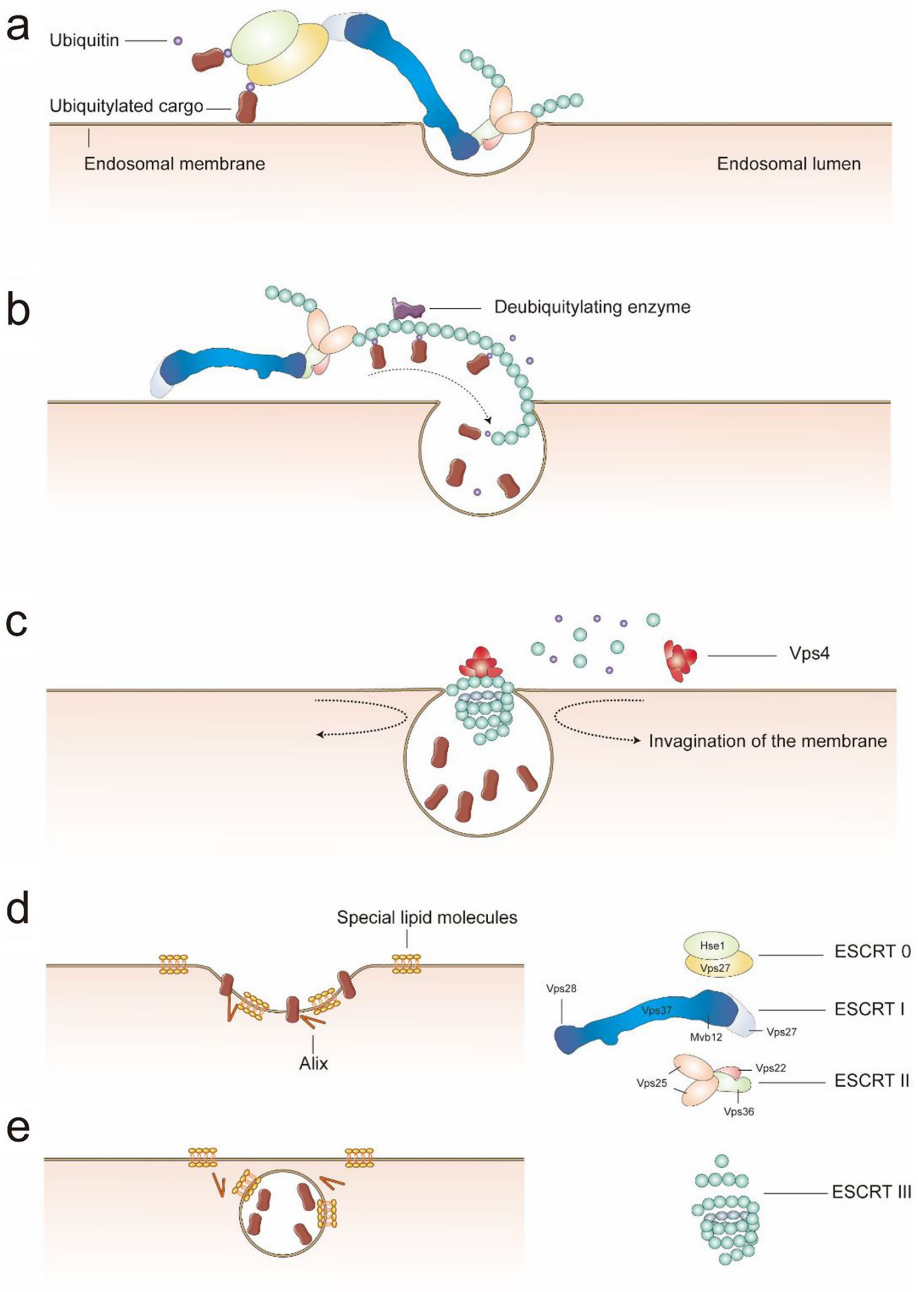
The ESCRT-dependent pathway (**a**-**c**) and ESCRT-independent pathway (**d**,**e**) in protein sorting. (**a**) The recognition and sorting of ubiquitinated proteins mediated by ESCRT complexes. This figure highlights the way ESCRT complexes interact and function together. (**b**) This figure illustrates how cargo proteins are sorted into ILVs, and the transforming of ESCRT-III to a helical filament structure that aids in membrane invagination and the entry of proteins. (**c**) The endosomal membrane further invaginates during the ESCRT-III helical process, and the Vps4 protein participates in the disassembly and recycling of the ESCRT-III complex. (**d**, **e**) In the ESCRT-independent pathway discovered in mammalian cells, ILV formation and protein transport can still occur after the silencing of key subunits of ESCRT proteins. This process primarily relies on the interactions between membrane lipids (such as ceramide and LBPA) and intracellular proteins (such as Alix)

In the process of a typical ESCRT-dependent pathway, the recognition of ubiquitylated proteins is initially mediated by ESCRT-0, which consists of the subunits Hrs and STAM (known as Vps27 and Hse1 in yeast). ESCRT-0 participates in recruiting ESCRT-I, and the recruitment of ESCRT-I to endosomal membranes will be impeded without ESCRT-0 [30]. Crystallographic study on yeast has confirmed the structure of ESCRT-I with four subunits: Vps23, Vps28, Vps37, and Mvb12. The endpiece of ESCRT-I which contains the ESCRT-0-binding domain contributes to the recruitment of ESCRT-I to the membrane. The characteristic structure of ESCRT-I is the long stalk domain which is essential for the correct disposition of cargo [31]. ESCRT-II is composed of four subunits: one Vps22, one Vps36, and two Vps25. It interacts with the subunit of ESCRT-I Vps28 through the GLUE (GRAM-like ubiquitin-binding in Eap45) domain of Vps36 [32], and binds with ESCRT-III through the subunit Vps25. ESCRT-III is composed of multiple small and highly charged subunits and is mainly recruited by ESCRT-II. ESCRT-III can assemble into filamentous oligomers which can further transform into helical tubes or conical funnels to enable the attachment and entrance of cargo to the invaginations of membranes. The inverse budding into MVBs is mediated by ESCRT-III and Vps4 through the process of plasma-membrane abscission. Currently, the mechanism of the scission process remains unclear, one model explains this process as the strong binding between the helix ESCRT-III complex and the lipid membrane causes the vesicle neck to contract, leading to vesicle fission [33]. Before the sorting of proteins is finished, deubiquitylating enzymes are recruited by ESCRT-III to maintain the recycling of ubiquitin [34], and Vps4 as a kind of ATPase participates in dissociating ESCRT-III for the recycling of ESCRTs [35] (Fig. 2a-c).

The ESCRT-dependent pathway has been thoroughly researched in yeast, while in mammalian cells, ILVs can still form in the absence of ESCRT components [36] (Fig. 2d and e). This kind of ESCRT-independent pathway can be driven by the presence of lipid molecules along with essential proteins. Ceramide is the product of sphingomyelin hydrolysis by sphingomyelinases. In mouse oligodendroglial cells, researchers revealed that the release of exosomes decreased by inhibiting sphingomyelinases. Furthermore, sphingomyelinases can increase the budding of small vesicles from the giant unilamellar vesicles model [37]. Lysobisphosphatidic acid (LBPA), a kind of lipid molecule that is abundant in the endosomal membrane, can induce the creation of membrane invaginations in acidic liposomes by interaction with Alix [38]. Currently, researchers are able to analyze the complete lipid and protein components of the membrane, but functional studies of specialized cellular membrane regions remain a challenge. Evidence suggests that although ESCRTs can ensure the efficiency and accuracy of protein sorting, they are not necessarily required for the formation of ILVs, and their specific functional region within this context is not indispensable [26].

To characterize exosomes, it is essential to demonstrate from the perspectives of structural identification and qualitative detection of biomarkers. The observation of exosomes requires microscopes with sufficiently high resolution. Transmission electron microscopy (TEM), with a resolution that can reach 0.1 ~ 0.2 nm, enables the visualization of exosomes featuring distinct lipid bilayers, alongside a unique cup-shaped structure [39]. In recent years, nanoparticle tracking analysis (NTA) has been increasingly utilized for exosome detection. NTA tracks and analyzes observed particles, ultimately providing analysis results of particle size distribution and particle concentration. It is possible to statistically analyze the size and quantity of exosomes, as well as perform preliminary quality assessment [40–42].

In addition to morphology detection, exosomes possess unique protein biomarkers that offer characteristics for their identification. The most commonly detected proteins from exosomes are the tetraspanin family (mainly refers to CD9, CD63, CD81, and CD82), which can be demonstrated in various studies focusing on HCC. Other commonly detected marker proteins include membrane transport proteins (Rab GTPases and Annexins), heat shock proteins (HSPA8 and HSP90), Alix, and TSG101 [43] (Fig. 1). In one pan-cancer analysis of EVs from 426 human samples, CD9, HSPA8, Alix, and HSP90 were identified as the most prominent markers. In addition, ACTB, MSN, and RAP1B can serve as novel pan-EV markers [44].

Functionally, research has revealed that the overexpression of CD9 and CD81 can inhibit HCC cell proliferation through the Krüppel-like factor 4 (KLF4)-CD9/CD81-Jun N-terminal kinase (JNK) signaling pathway. Changes in the levels of CD9 and CD81 do not impact the expression of exosomal CD63, Alix, and TSG101 [45]. Another exosomal marker CD63 was discovered as a kind of sialoglycoprotein, and the glycosylation of CD63 mediated by silencing α2,6-sialyltransferase I (ST6Gal-I) can alleviate the effects of HCC-derived EVs in promoting tumor progress, mainly through blocking the Akt/Glycogen synthase kinase (GSK)-3β or JNK1/2 pathways [46]. Although EVs still possess many other characteristic markers, including lipids and glycoproteins, researchers are increasingly focused on functionally significant molecules and proteins, including cargo contained within them.

### The role EV-associated proteins play in HCC

**Fig. 3:**
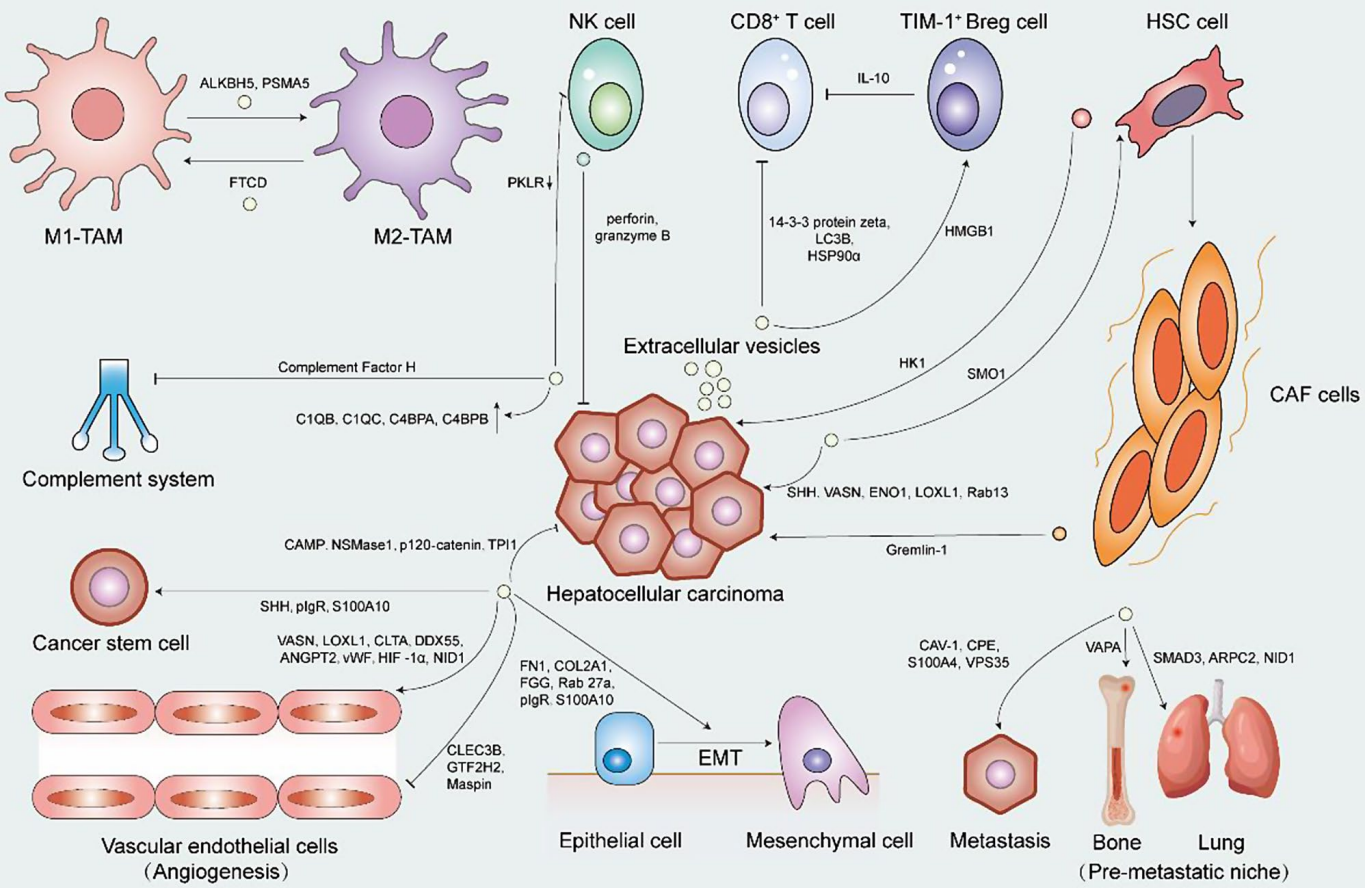
The role EV-associated proteins play in HCC. The interactions between HCC cells and TME components, including TAM, NK cell, CD8^+^ T cell, TIM-1^+^ Breg cell, HSC cell, CAF cell, and cancer stem cell, and essential processes in the development of HCC including angiogenesis, EMT, metastasis, and the formation of the pre-metastatic niche based on EV-associated proteins are summarized. EVs with different colors represent different cellular origins

EVs carry a variety of proteins that participate in the regulation of HCC. During the progression of HCC, EVs produced by tumor cells and stromal cells in the TME serve as communication tools, capable of altering the proliferation, metabolism, phenotype, and function of recipient cells. As HCC progresses, tumor cells exhibit a tendency for metastasis, often targeting specific sites, which involves processes including angiogenesis, reacquisition of tumor stemness, and epithelial-mesenchymal transition (EMT). The study of EV-associated proteins holds significant importance in the understanding of the tumor and microenvironment characteristics of HCC, tumor metastasis mechanisms, and their potential prospects in HCC treatment. In Fig. 3, we provide an overview of the functions of different EV-associated proteins in the context of HCC development and progression.

### Tumor progression and TME

The progression of HCC not only depends on its biological characteristics but is also closely associated with its surrounding tumor microenvironment. The tumor microenvironment refers to the complex environment composed of tumor cells and the surrounding stromal cells and matrix components [47]. In this intricate environment, studies on EV-associated proteins mainly involve HCC cells, intrinsic immune cells, adaptive immune cells, hepatic stellate cells (HSCs), and cancer-associated fibroblasts (CAFs) among others.

Researchers have observed that EVs derived from HCC cells harbor a variety of proteins, some of which can inhibit tumor growth while others can promote tumor progression. Proteins with tumor growth inhibitory functions act through multiple pathways. Through cell cycle analysis, researchers have observed that elevated levels of cathelicidin antimicrobial peptide (CAMP) are associated with reduced cell proliferation and a significant delay in the G1-S transition. This phenomenon was observed to be diminished in the circulating EVs of HCC patients [48]. Neutral sphingomyelinase 1 (NSMase1) within EVs secreted from HCC cells, which can convert sphingomyelin to ceramide, is able to inhibit cell growth and induce apoptosis of HCC cells via reducing the ratio of sphingomyelin/ceramide [49]. Another EV-derived protein, p120-catenin, secreted from HCC cells can suppress the growth and progression of HCC cells by inhibiting the signal transducer and activator of transcription (STAT) pathway [50]. Besides inhibiting tumor growth, emerging evidence suggests proteins contained in HCC-associated EVs also possess functions that promote tumor growth. A kind of hedgehog protein in EVs, sonic hedgehog (SHH), is revealed to promote HCC progression through the SHH pathway and facilitate the formation of cancer stem cells (CSCs) [51]. Another transmembrane glycoprotein expressed in EVs from HCC cells, Vasorin (VASN), is reported with pro-angiogenic functions and can promote tumor cell proliferation and migration through activation of the STAT3 signaling pathway [52, 53].

Tumor cells exhibit a preference for glycolysis in the TME, which is a metabolic pathway to maintain an elevated growth rate. Even when oxygen is abundant, tumor cells prefer aerobic glycolysis to mitochondrial oxidative phosphorylation. This process is known as the Warburg effect. For HCC, Alpha-enolase (ENO1) is an essential enzyme for glycolysis that contributes to the lactic acid production in tumor cells. EV-derived ENO1 can upregulate integrin α6β4 expression and activate the focal adhesion kinase (FAK)/Src/p38 pathway to promote tumor growth and metastasis of HCC cells [54]. Another protein from EVs, triose-phosphate isomerase 1 (TPI1), as a kind of homodimer glycolytic enzyme that participates in the glycolysis, is found to decrease the aerobic glycolysis in the recipient HCC cells. The decreased levels of EV-TPI1 enhance aerobic glycolysis-driven tumorigenesis. Furthermore, the level of TPI1 in EVs is positively correlated with the level of Rab27 in HCC cells, which is often downregulated in HCC [55].

In the TME of HCC, EVs serve as mediators for intercellular communication. These EVs can originate from HCC cells themselves or from surrounding stromal and immune cells. Investigating the functions of proteins within these EVs provides crucial insights into the developmental patterns of the TME. Among numerous proteins, lysyl oxidase-like 4 (LOXL4) has garnered considerable research attention. LOXL4 is a member of the lysyl oxidase family, exhibiting multiple tumorigenic effects. It can spread among HCC cells via EVs, facilitating tumor metastasis through the FAK/Src pathway and promoting angiogenesis [56]. In addition, LOXL4 shuttled by EVs can induce the expression of programmed death ligand 1 (PD-L1) on macrophages and immunosuppression by activating the STAT1/PD-L1 pathway, thus promoting an immunosuppressive microenvironment and inducing the immune escape of HCC [57, 58].

For the innate immunity of HCC, tumor-associated macrophages (TAMs) play a crucial role in immune evasion and are a focal point of investigation. TAMs are one of the most common stromal cells in the TME of HCC which derive primarily from circulating monocytes. Research reveals that HCC cells can promote monocyte-to-macrophage differentiation through the pyruvate kinase M2 isoform (PKM2)-dependent manner by ectosomes. The reshaped monocytes/macrophages can further secrete cytokines to promote the proliferation of HCC cells [22]. Typically, macrophages can be divided into two subtypes: the classical M1 and the alternative M2 macrophages. In the early stage of tumors, TAMs mainly exhibit the M1 phenotype to inhibit angiogenesis and promote immunity. As the tumor progresses, the tumor microenvironment typically induces polarization from the M1 phenotype towards the M2 phenotype. M2 macrophages possess a limited antigen-presenting capacity and can promote angiogenesis, enhance tumor cell invasion, and inhibit T-cell immune responses by releasing immunosuppressive factors IL-10 and TGF-β [59, 60]. Some studies have revealed the mechanisms that EV-associated proteins contribute to TAM polarization. AlkB homolog H5 (ALKBH5) is a kind of N^6^-methyladenosine (m^6^A) demethylase. Elevated levels of ALKBH5 can promote HCC cell stemness and are associated with poor prognosis, mainly through activating the SOX4/SHH signaling axis. EVs originating from HCC cells have the potential to transfer ALKBH5 to THP-1 cells (a kind of human monocytic cell), which is related to macrophage M2 polarization [61]. EVs containing proteasome subunit alpha 5 (PSMA5) possess similar functions in promoting M2 polarization of macrophages, mainly by activating Janus Kinase 2 (JAK2)/ STAT3 pathway [62]. Conversely, EVs from HCC cells containing formimidoyltransferase-cyclodeaminase (FTCD) have the ability to promote macrophage polarization towards M1 and suppress the proliferation of HCC cells [7]. To further suppress the M2 phenotype conversion, researchers have engineered EVs by conjugating antisense oligonucleotides (ASOs) to the prostaglandin F2 receptor negative regulator (PTGFRN) on the EV membrane. This approach effectively silences the expression of STAT6, a critical transcription factor involved in M2 polarization, reshaping the tumor microenvironment of HCC. Consequently, it promotes the polarization of M1-type TAMs and inhibits HCC growth [63].

Natural killer (NK) cells can produce EVs containing cytotoxic proteins to kill tumor cells. Research indicates that stimulating NK cells with IL-15 and IL-21 can lead to the production of EVs containing perforin and granzyme B, enhancing cytotoxicity and apoptosis of HCC cells [64]. Another research illustrates that NK cell-derived EVs can exert potent anti-tumor effects by inhibiting serine/threonine kinase pathway-associated cell proliferation and enhancing caspase activation pathway-associated apoptosis [65]. Correspondingly, HCC cells can also influence the function of NK cells through EVs. One characteristic of HCC cells is the suppression of gluconeogenic function. This leads to the secretion of pyruvate kinase (PKLR)-attenuated EVs, which can inhibit the function of NK cells, thereby promoting the tumorigenic process [66].

In the TME of HCC, tumor-infiltrating T lymphocytes (TILs) play a pivotal role in adaptive immunity. CD8^+^ CTLs specifically inhibit tumor growth by killing tumor cells through cytotoxicity. Mounting evidence suggests that EVs produced by HCC predominantly exert inhibitory effects on the function of CD8^+^ CTLs. High expression of 14-3-3 protein zeta in both HCC cells and CD8^+^ TILs can facilitate the proliferation, EMT of HCC cells and CD8^+^ TILs exhaustion. It is suggested that 14-3-3 protein zeta may be transferred from HCC cells to CD8^+^ TILs, potentially via EVs, contributing to these effects [4]. The B isoform of microtubule-associated protein 1 light chain 3 (LC3B) functions as an EV marker. Evidence suggests that LC3B^+^ EVs hinder the immune response by inducing inflammation. These EVs stimulate leukocytes to secrete IL-6 and IL-8 by transporting HSP90α. IL-6 and IL-8 contribute to the suppression of CD8^+^ T cell function, thus impacting the effectiveness of immunotherapy. While blocking HSP90α from LC3B^+^ EVs has the potential to improve the effectiveness of anti-PD-1 treatment. This provides a new perspective for enhancing the response rate of immunotherapy for HCC [67, 68].

Breg cells as a subset of B cells, contribute to immune modulation and suppress immune responses in HCC. T cell Ig and mucin domain (TIM)-1^+^ Breg cells as a subgroup of Breg cells, can impair the functions of CD8^+^ T cells and accelerate HCC progression by producing abundant IL-10. EVs containing high mobility group box 1 (HMGB1) released by HCC cells can enhance the accumulation of TIM-1^+^ Breg cells via the toll-like receptor (TLR) 2/4 and mitogen-activated protein kinase (MAPK) pathway [69]. This suggests that EVs derived from HCC primarily promote the proliferation of Breg cells and accelerate tumor progression.

HSCs are resident mesenchymal cells that can be activated in response to liver injury and participate in the formation of liver fibrosis. In addition, HSCs are a significant contributor to CAFs [70]. Both HSCs and CAFs primarily serve to promote tumor progression. In the TME of hepatic fibrosis, hexokinase 1 (HK1) secreted from HSCs via large EVs can be captured by HCC cells and can promote tumor glycolysis and progression. In addition, the small molecule PDNPA disrupts Akt-mediated degradation of Nur77, resulting in reduced release of HK1. This finding holds promise for inhibiting HCC progression [71]. On the other hand, HCC cells can actively produce EVs to activate HSCs, ultimately promoting tumor development. EVs derived from HCC cells transmit SMO, a key signal transducer in the Hedgehog pathway, to HSCs, leading to the proliferation, invasion, migration, and EMT of HSCs, and further accelerating HCC development in a positive feedback manner [72]. CAFs promote tumorigenic features by remodeling the extracellular matrix to facilitate tumor proliferation, metastasis, angiogenesis, and drug resistance [73]. EVs secreted from CAFs containing Gremlin-1 can induce EMT of hepatoma cells and induce resistance to sorafenib, possibly through activation of the Wnt/β-catenin pathway [74]. Additional research has also revealed tumor-specific communication between HCC cells and CAFs. EVs isolated from HCC cells can stimulate the phospho-extracellular regulated protein kinases (pERK)1/2 signaling and upregulate mitogen-activated protein kinase (MAPK) and Wnt in fibroblast cells, while EVs from fibroblast cells can notably increase the levels of SPOCK1 (also known as testican-1), a proteoglycan recognized as oncogenic, in HCC cells [75].

### Angiogenesis

Angiogenesis is a characteristic feature of tumor growth, and hypoxia is often considered to strongly stimulate tumor angiogenesis. At the molecular level, upregulated expression of pro-angiogenic factors such as vascular endothelial growth factor (VEGF) and platelet-derived growth factors (PDGF) in the TME can promote angiogenesis by activating the phosphatidylinositol-3 kinase (PI3K)/Akt/mTOR pathway [76]. Proteins from EVs have been found to widely participate in this process.

The imaging of angiogenesis has confirmed that the number of EVs secreted from HCC cells could affect the lumen formation of human umbilical vein endothelial cells (HUVECs) [77]. Many proteins have been found to have pro-angiogenic functions. Clathrin light chain A (CLTA) is one subunit of the light chain of clathrin. It is found overexpressed in EVs of HCC and can enhance angiogenesis and break up the integrity of vascular endothelial barriers through the stabilization of basigin. The inhibitor of basigin can inhibit patient-derived xenografts (PDXs) tumor progression in mice [78]. DEAD-box helicase 55 (DDX55) is a member of the DEAD‐box RNA helicase family related to early recurrence of HCC after surgery. EVs containing DDX55 from HCC cells can enhance the proliferation and tube formation abilities of endothelial cells, thus stimulating angiogenesis and enhancing the malignancy of HCC cells [79]. Angiopoietin-2 (ANGPT2) plays a significant role in promoting tumor angiogenesis and inflammation. EVs containing ANGPT2 participate in the transportation from HCC cells to HUVECs, thereby enhancing angiogenesis notably [5]. One kind of EV-derived protein, von Willibrand factor (vWF) can upregulate the levels of VEGF-A and fibroblast growth factor 2 (FGF2) in endothelial cells to promote angiogenesis, and FGF2 can promote HCC cell growth in a positive feedback manner by activating the FGFR4/ERK1 signaling pathway [80]. Similar findings including EVs containing hypoxia-inducible factor (HIF) -1α can promote angiogenesis through the PI3K/Akt/mTOR pathway [81], and another EV-derived protein Rab13 can promote angiogenesis by upregulating VEGF in HCC cells [82], further demonstrated the positive effects of EV-associated proteins on angiogenesis.

Contrary to the above findings, researchers also revealed EVs containing proteins with anti-angiogenesis functions. C-Type Lectin Domain Family 3 Member B (CLEC3B) is a transmembrane Ca^2+^-binding protein expressed on EVs. High levels of CLEC3B can inhibit tumor migration and angiogenesis by targeting the VEGF pathway and inhibiting the EMT process. This suggests that VEGF-targeting therapy for HCC patients may benefit from CLEC3B-high EVs [83]. Another EV-derived protein secreted by HCC cells, general transcription factor II subunit H2 (GTF2H2), can inhibit the migration and permeability of HUVECs and thus suppress tumor angiogenesis [84], where the result needs further confirmation in animal models. Another study has demonstrated that HCC cells exposed to radiation can produce EVs rich in Maspin, which is regulated by histone deacetylase 5 (*HDAC5*). These EVs can inhibit angiogenesis, providing a new perspective for enhancing the sensitivity of radiotherapy [85]. Currently, multi-kinase inhibitors or anti-angiogenic drugs targeting the VEGF/VEGFR signaling pathway, such as sorafenib, lenvatinib, and bevacizumab, have been used in first-line and second-line treatments for HCC. However, due to the limited response rates of these drugs, exploring more effective therapies from the perspective of EVs holds promise.

### Tumor metastasis

In the advanced stages of HCC, there is often a tendency for targeted organ metastasis, with lung metastasis being the most common, accounting for over 70% of HCC-related deaths [86]. This specific metastatic pattern relies on the formation of pre-metastatic niches, where the activation of EMT is considered a crucial mechanism for metastasis initiation. Research indicates that EVs play a widespread role in the process of HCC metastasis.

EMT is a biological process where epithelial cells lose their structural organization, including polarity and cell-to-cell junctions, and acquire a mesenchymal phenotype, boosting their migratory and invasive capabilities. The EMT process is typically accompanied by the increased expression of α-SMA and vimentin, and decreased expression of E-cadherin [87]. Studies suggested that EVs can promote the invasion and metastasis of HCC cells by inducing EMT. EVs from the highly metastatic MHCC97H cells can induce the low metastatic MHCC97L cells to undergo EMT through the MAPK/ERK pathway, and the levels of Rab27a from HCC cells influence the secretion levels of EVs [88]. Compared to complete EMT, HCC cells undergoing partial EMT retain both epithelial and mesenchymal characteristics to some extent. Early identification of the EMT process can be achieved through EV-associated proteins serving as biomarkers. Researchers revealed that enhanced secretion levels of post-translationally modified fibronectin 1 (FN1), collagen type II alpha 1 (COL2A1), and native fibrinogen gamma chain (FGG) in EVs can serve as biomarkers for chemo-resistance and partial EMT [89].

In addition to participating in the EMT process, proteins from EVs can also promote metastasis by boosting the stemness of tumor cells. Polymeric immunoglobulin receptor (pIgR) enriched in EVs from HCC cells can promote cancer stemness and aggressiveness by inducing the Akt/ β-catenin axis rather than activating the SMAD2/3 signaling and inducing EMT in HCC cells. In addition, the neutralizing of EV-pIgR with antibodies can provide an option for the treatment of HCC [90]. Another EVs-loaded protein S100A10 is found to enhance the stemness characteristics of HCC, promote pulmonary leakiness and EMT, and enhance HCC progression through epidermal growth factor receptor (EGFR) activation [91].

Based on inducing tumor stemness and EMT that promote tumor cell dissemination, EV-associated proteins play a crucial role in guiding “seed” to specific “soil”. The “seed and soil” hypothesis indicates that tumor cells (seeds) disseminate throughout the body but only grow in specific organ microenvironments (soil), and this underscores the importance of highlighting the significance of the pre-metastatic niche. Research suggests that EVs from HCC cells can promote lung metastasis formation by regulating circulating tumor cells (CTCs) proliferation and adhesion by releasing SMAD family member 3 (SMAD3) protein [92]. Actin-related protein 2/3 complex subunit 2 (ARPC2) is highly expressed in EVs of metastatic HCC cells, and ARPC2 inhibitor Pimozide can suppress the colonization of metastatic cells in the lung [93]. One EVs-loaded protein nidogen 1 (NID1) can enhance the permeability of pulmonary endothelial cells, promote angiogenesis, and increase the expression of tumor necrosis factor receptor 1 (TNFR1), to facilitate the formation of a pre-metastatic niche in the lung, thereby promoting lung metastasis of HCC [94]. Another research reveals that the levels of transmembrane serine protease 2 (TMPRSS2) in tumors are conversely correlated with the NID1 levels in EVs, indicating that inactivating TMPRSS2 can counteract the malignant characteristics of HCC [95]. For the bone metastasis of HCC, new evidence indicates that a transmembrane protein of EVs, VAMP-associated protein A (VAPA), can be recognized by osteoclast membranes and promote their activation, thereby fostering a fertile niche conducive to the growth of HCC cells [96].

Some other studies have explored the impact of EV-associated proteins on HCC metastasis from various perspectives, involving the complement system, reciprocal induction of cell transmigration, and drug-related events. In the complement system, complement Factor H (CFH), a kind of soluble protein abundant in EVs of metastatic HCC cell lines that can inhibit the alternative complement pathway, is capable of reducing complement-mediated cell lysis. By inhibiting the complement system to evade immune surveillance, the invasive ability of HCC cells can be enhanced [97]. In another proteomic quantitative analysis study involving 21 HCC patients and 15 healthy controls, a series of complement-related proteins were identified and validated to be upregulated in HCC patients, including complement C1Q subcomponent subunit B (C1QB), complement C1Q subcomponent subunit C (C1QC), C4B-binding protein alpha chain (C4BPA), and C4B-binding protein beta chain (C4BPB), further elucidating the role of the complement activation pathway in the tumorigenesis of HCC [98].

Caveolin-1 (CAV-1) as a structural protein of caveolae, is found highly-expressed in the EVs derived from metastatic HCC cell lines and can enhance the metastatic and invasive ability of immortalized hepatocytes [99, 100]. Carboxypeptidase E (CPE) is an exopeptidase upregulated in EVs from HCC cells. The proliferation and invasion ability of low-metastatic potential MHCC97L cells can notably enhance in the CPE-dependent pathway after incubation together with EVs derived from MHCC97H cells with high-metastatic potential [6]. In addition, EV-derived protein S100A4 has also been found to have similar intercellular crosstalk functions to promote the metastatic potential of MHCC97L cells [101].

There is also a drug-related event promoting tumor metastasis reported, where STA9090, as an inhibitor of HSP90, poses a risk of promoting HCC metastasis. Vacuolar protein sorting-associated protein 35 (VPS35) is found to be upregulated in the EVs from HCC cells when stimulated by STA9090 and can enhance tumor metastasis, primarily through the activation of the Bclaf1-VPS35-EVs axis [102].

### The application of EV-associated proteins as diagnostic and therapeutic biomarkers for HCC

HCC diagnosis is based mainly on its clinical and imaging characteristics and is approved by guidelines. While similar imaging features, such as between HCC and intrahepatic cholangiocarcinoma (ICC), can result in misidentification and the implementation of inappropriate treatment strategies [103]. At present, there is limited consensus in clinical practice regarding the routine biopsy of newly detected liver tumors. Consequently, investigating non-invasive or minimally invasive liquid biopsy methods to identify dependable biomarkers holds promise for enhancing the diagnostic precision of HCC. Proteins within EVs serve as markers and can offer distinct advantages over traditional blood-based markers. Derived from parent cells and enclosed within protective lipid bilayers, EV proteins can provide increased stability and specificity for tumor diagnosis [10]. Notably, it should be noted that the sensitivity of minimally invasive liquid biopsy methods for detecting early-stage HCC may be constrained. This limitation could stem from the necessity for the tumor to advance to a vascularized stage before a substantial quantity of detectable EVs is generated [104].

Glypican-3 (GPC-3) is a heparin sulfate proteoglycan, typically located on the cell membrane of the fetal liver cells, but also expressed in malignant tumors, such as HCC and lung carcinoma [105]. Research indicates that GPC-3 is an optimal target for EV-based surveillance of HCC. GPC-3 is predominantly found within EVs, with only a minor presence of its soluble form in the serum. GPC-3 derived from EVs performs better than AFP in discriminating HCC patients from cirrhotic patients and healthy controls [106]. It can also be detected in patients with cirrhosis, and increased expression found in EVs is associated with impaired hepatocellular autophagy [107]. Additionally, as a membrane protein, GPC-3 can also be purified and analyzed through antibody selection using fluorescence nanoparticle tracking analysis (F-NTA) and is positively correlated with the total tumor size of HCC [108]. As a widely recognized EV membrane protein, in terms of treatment, GPC-3 single-chain scFv antibody can be utilized to target the induction of EVs loaded with IR780 and Lenvartinib, enhancing the efficacy of hyperthermia and chemotherapy [19].

EVs with specific protein expression profiles can be identified to recognize patients with HCC. Through fluorescence-activated cell scanning (FACS), AnnexinV^+^ EpCAM^+^ ASGPR1^+^ EVs are identified as potential biomarkers to distinguish between HCC patients and cirrhotic patients without detectable tumors, with an area under the curve (AUC) of 0.732 and exhibit a notable decrease by the seventh day following surgery [104]. Chen et al. also discovered HSP90α^+^ LC3B^+^ EVs as potential biomarkers to distinguish HCC patients from non-liver cancer controls (AUC = 0.960). The research further revealed a positive correlation between the levels of HSP90α^+^ LC3B^+^ EVs and PD-1^high^ CD8^+^ exhausted T cells [68].

In recent years, researchers have further employed the use of tissue microarray methodology to aid in the quantitative analysis of EV subpopulations. An HCC EV ECG score, derived from the measurements of three distinct HCC EV subpopulations (EpCAM^+^ CD63^+^, CD147^+^ CD63^+^, and GPC3^+^ CD63^+^), was developed to detect early-stage HCC. In the validation cohort, the AUC of the ECG score for distinguishing early-stage HCC from cirrhosis reached 0.93 [109].

Through proteomic analysis of HCC cell lines followed by validation in clinical cohorts, one study identified two EV-enriched proteins, CCT8 and cofilin-1. These proteins demonstrate promise as serum biomarkers for diagnosing HCC, exhibiting respective AUC values of 0.698 and 0.677, notably surpassing that of AFP (AUC = 0.63). The combined utilization of all three biomarkers achieved the highest AUC (AUC = 0.84) [110].

The detection of tumor-associated (TA) autoantibodies holds promise for early tumor identification, and their secretion via EVs has been observed. Studies have identified several antigens recognized by TA autoantibodies, including ATIC, bromodomain-containing protein 2 (BRD2), and translation initiation factor 3 subunit A (EIF3A). These TA autoantibodies demonstrate effective discrimination between serum samples from HCC patients and healthy controls and are considered potential diagnostic biomarkers within EVs [111–113].

Other studies have identified several candidate protein biomarkers for HCC diagnosis or prognosis in EVs either within limited cohorts or solely validated using bioinformatics methods [114], including, haptoglobin (HP), transthyretin (TTR) [115], kinesin family member 2 C (KIF2C), targeting protein for xenopus kinesin-like protein 2 (TPX2) [116], LAPTM4B-35 [117], two isoforms MRP3A and MRP3B of ATP Binding Cassette Subfamily C Member 3 (ABCC3) [118], vWF together with other nine candidate proteins [119]. However, comprehensive explorations of their diagnostic efficacy remain unexplored.

In comparison to the blood proteome, the urine proteome is less complex, thus making it easier to detect changes in low-abundance proteins that may have potential significance. Currently, there is limited research focused on the proteomics of urinary EVs (uEVs). In an article published in 2023, Feng et al. reported the significant enrichment of three proteins, olfactomedin 4 (OLFM4), growth differentiation factor 15 (GDF15), and hepatocellular carcinoma-derived growth factor (HDGF), in uEVs from patients with HCC using an array-based amphiphilic supramolecular probe (ADSP)-modified NC membrane platform [120]. Another study explored the glycoproteomic profile of uEVs, focusing on the post-translational modification of proteins. The results revealed significantly elevated levels of galectin-3-binding protein (LG3BP), polymeric immunoglobulin receptor (pIgR), and kininogen-1 (KNG1) glycosylation in patients with HCC compared to the control group. Conversely, the level of apoptosis stimulating of p53 protein 2 (ASPP2) was found to be decreased [121]. However, further validation of the diagnostic efficacy of these studies is still needed.

EV-based proteomics has also been employed to predict treatment response. In a clinical trial involving 25 patients receiving selective internal radiation therapy (SIRT) plus sorafenib treatment and 20 patients receiving sorafenib alone for advanced HCC, high levels of EV-GPX3/ACTR3 and low levels of EV-ARHGAP1B were associated with greater efficacy of SIRT plus sorafenib treatment (AUC = 1) [122]. This encouraging result suggests that exploring EV-based biomarkers in a clinical trial setting is a promising avenue for future research.

Some other studies have revealed potential EV-associated protein markers, which await further exploration. One study indicated the RNA level of splicing factor 3b subunit 4 (SF3B4) derived from EVs can serve as a diagnostic biomarker for HCC, with the AUC surpassing that of AFP. However, further validation is essential to ascertain the protein expression levels of *SF3B4* within EVs [123]. Another study has demonstrated the presence of tissue transglutaminase 2 (TGM2) protein, unique to HCC cell-derived EVs, suggesting its potential as a candidate diagnostic biomarker for HCC [124]. In order to display the existing research on EV-associated proteins in the field of HCC biomarkers, we listed Table 1 with key information to offer a concise and informative overview.

**Table 1:** The current status of research on EV-associated proteins as biomarkers for HCC

Biomarkers	Cohort number	AUC	Se/Spe (%)	PPV/NPV (%)	Applications	Ref.
AnnexinV^+^EpCAM^+^ASGPR1^+^	HCC: 86 Cirrhosis: 49	0.732 (0.646–0.818)	81.40/41.94	72.92/58.97	Diagnosis of HCC in the background of cirrhosis	[104]
EpCAM^+^ CD63^+^/ CD147^+^ CD63^+^/ GPC3^+^ CD63^+^	HCC: 35 Cirrhosis: 37 (Validation cohort)	0.93 (0.87–0.99)	91/81	82/91	Diagnosis of HCC in the background of cirrhosis	[109]
CCT8	HCC: 132 Cirrhosis: 33 CHB: 25 Normal: 34	0.698 (0.634–0.758)	46.21/93.48	58.93/91.05	Diagnosis of HCC	[110]
cofilin-1	HCC: 132 Cirrhosis: 33 CHB: 25 Normal: 34	0.677 (0.612–0.738)	40.91/94.57	91.53/52.73	Diagnosis of HCC	[110]
CCT8 + cofilin-1 + AFP	HCC: 132 Cirrhosis: 33 CHB: 25 Normal: 34	0.838 (0.783–0.884)	70.46/81.52	84.55/65.79	Diagnosis of HCC	[110]
HSP90α^+^ LC3B^+^	HCC: 51 NMLD: 33 Normal: 30	0.960 (NA)	86.00/96.67	NA	Diagnosis of HCC	[68]
High GPX3/ACTR3 Low ARHGAP1B	HCC: 45	1.0 (NA)	NA	NA	Predicting the efficacy of SIRT + sorafenib therapy	[122]
Anti-ATIC	HCC: 144 Normal: 118	0.875 (0.834–0.917)	70.83/90.63	NA	Diagnosis of HCC	[111]
Anti-BRD2	HCC: 118 Cirrhosis: 32 Benign: 3 Normal: 91	0.776 (0.714–0.839)	64.41/82.42	NA	Diagnosis of HCC	[112]
Anti-EIF3A	HCC: 102 Normal: 85	0.871 (0.822–0.922)	79.41/83.53	NA	Diagnosis of HCC	[113]
GPC-3	HCC: 25 Cirrhosis: 25 Normal: 25	1.0 (NA) (vs. normal) 0.95 (NA) (vs. cirrhosis)	NA	NA	Diagnosis of HCC	[106]
MRP3A/MRP3B	HCC: 14 Cirrhosis: 27 Normal: 15	NA	NA	NA	Higher expression in EVs from HCC	[118]
LAPTM4B-35	HCC: 43 Normal: 33	NA	NA	NA	Higher expression in EVs from HCC	[117]
HP	HCC: 15 Cirrhosis: 15 CHB: 15	NA	NA	NA	Higher expression in EVs from HCC compared to cirrhosis and CHB	[115]
TTR	HCC: 15 Cirrhosis: 15 CHB: 15	NA	NA	NA	Lower expression in EVs from HCC compared to CHB	[115]
vWF/ LGALS3BP/ TGFB1/ SERPINC1	HCC: 20 Normal: 10	NA	NA	NA	Higher expression in EVs from HCC	[119]
HPX/ HP/ HBA1/ FGA/ FGG/ FGB	HCC: 20 Normal: 10	NA	NA	NA	Lower expression in EVs from HCC	[119]
COL1A2/ POSTN/ STAM/ COL6A1/ EXOC8	HBV-related HCC cell lines Normal liver cells	NA	NA	NA	Higher expression in EVs from HBV-related HCC cells	[114]
KIF2C/ TPX2	HBV-related HCC: 225 Normal: 220	NA	NA	NA	Higher expression in EVs from HCC with poor OS and RFS	[116]
TGM2	NA	NA	NA	NA	Unique in the EVs from HCC cells	[124]
OLFM4/ GDF15/ HDGF	HCC: 18 Normal: 6	NA	NA	NA	Higher expression in uEVs from HCC	[120]
LG3BP/ pIgR/ KNG1	HCC: 21 Normal: 7	NA	NA	NA	Higher expression in uEVs from HCC	[121]
ASPP2	HCC: 21 Normal: 7	NA	NA	NA	Lower expression in uEVs from HCC	[121]

Se, sensitivity; Spe, specificity; PPV, positive predictive value; NPV negative predictive value; Ref., reference; CHB, chronic hepatitis B; HBV, hepatitis B virus; NMLD, non-malignant liver disease; OS, overall survival; RFS, relapse-free survival; NA, not available

### The exploration of EV-associated proteins in HCC treatment

The current treatment methods for HCC include surgical treatment, chemotherapy, radiotherapy, targeted therapy, immunotherapy, and other approaches [125]. HCC exhibits high heterogeneity, with a high postoperative recurrence rate. Early-stage HCC frequently lacks symptomatic manifestations, leading to diagnosis at an advanced stage. Furthermore, patients with advanced HCC face a paucity of targeted pharmacological interventions tailored to their specific condition. Based on the specific protein expression profile of EVs and the ability to transport cargo, EVs demonstrate a remarkable level of selectivity. This selectivity is achieved through surface ligands that target specific receptors, as well as inherent cargo sorting mechanisms, thus enabling accurate delivery to target cells while minimizing off-target effects. Furthermore, EVs-mediated drug delivery is characterized by low toxicity, low immunogenicity, and high engineering versatility [126]. EVs differ from exogenous synthetic drug carriers in that the latter often exhibit strong immunogenicity and are prone to interact with drug proteins thus garnering increasing research attention [127].

LAMP2 (lysosomal associated membrane protein 2) B, as an abundantly expressed protein on the membrane of EVs, has been widely employed in the engineering of EVs. This enables the EVs to target tissues or organs carrying the corresponding receptors [128]. The tumor-targeted nano-delivery system, formed by the fusion of the SP94 peptide and the N-terminal RNA recognition motif (RRM) at both ends of the LAMP2 platform, can enhance sorafenib-induced ferroptosis in HCC [129]. We observed that to enhance the efficacy of ferroptosis and reduce liver toxicity, researchers have engineered EVs to achieve these aims. Researchers transfected CD47-expressing plasmids onto EVs to evade macrophage phagocytosis and displayed much lower toxicity. Additionally, they loaded the membrane with the ferroptosis inducer Erastin and internally loaded the photosensitizer RB through sonication. The combination of chemo-photodynamic therapy displayed significant efficacy and safety [130].

The clustered regularly interspaced short palindromic repeats (CRISPR)–associated nuclease protein 9 (Cas9)–based genome editing holds tremendous therapeutic potential but exhibits characteristics such as easy degradation in the bloodstream and lack of tissue-specific uptake, hampering its efficacy. Whereas EVs provide the most efficient delivery method for CRISPR-Cas9, particularly for the delivery of Cas9 ribonucleoprotein (RNP), serving as an excellent carrier. Research indicates that through electroporation, Cas9 RNP can be transported into EVs derived from HSCs and subsequently taken up specifically by liver cells. Notably, Cas9 RNP targeting lysine acetyltransferase 5 (*KAT5*) can suppress the growth of HCC, which suggests that therapeutics involving the editing of other HCC-related genes hold vast potential for treatment [131].

Recently, several EVs-loaded drugs have been developed to stimulate tumor-specific immune responses and can serve as tumor vaccines. Dendritic cells (DCs) are believed to enhance antigen presentation and improve immunogenicity upon stimulation by EVs derived from HCC. High-mobility group nucleosome-binding protein 1 (HMGN1) can enhance DC maturation and activation and consistently elicit Th1 immune responses. By conjugating HMGN1 with CP05, an anchoring protein on EVs, HMGN1 can be more efficiently delivered systemically into cells. Research evidence has demonstrated that this approach activates DCs, remodels the tumor microenvironment, activates memory T cells, and exhibits promising therapeutic prospects [132]. Furthermore, the research team proposed a universal immunotherapeutic approach that does not require the identification of tumor antigens. By engineering EVs derived from DCs simultaneously loaded with P47, AFP, and HMGN1, complete eradication of tumors in situ was achieved in mice with HCC [16]. One kind of histone deacetylase inhibitor, MS-275, has been shown to upregulate tumor-specific antigens, thereby enhancing both specific and nonspecific anti-tumor immune responses. EVs from HCC cells treated with MS-275 showed increased expression levels of HSP70 and major histocompatibility complex (MHC) class I polypeptide-related sequence B (MICB). Previous research has demonstrated that HSP70 from tumor EVs can selectively activate NK cell activity, which is also consistent with the enhanced NK cell activity observed in this study [133, 134]. Another inhibitor of DNA methyltransferase, 5-Aza-2’-deoxycytidine (5-Aza-CdR), has been shown to increase the production of EVs and immune-related protein components in hepatoma cells, including human leukocyte antigen-I (HLA-I) and NY-ESO-1 protein [135]. These findings suggest promising approaches for developing tumor vaccine strategies against HCC.

Some therapeutic prospects have been proposed to address challenges in chemotherapy, immunotherapy, and radiotherapy for HCC from the perspective of EV-associated proteins. Research indicates that the use of chemotherapy drugs, especially resistant anticancer drugs, can significantly increase the levels of HSP in EVs released by HCC cells. This further induces the biological activity of NK cells, suggesting that immunotherapy based on resistant anticancer drugs holds promising prospects [17]. Although HCC is not sensitive to radioiodine therapy with I^131^, it can still enhance the iodine uptake capacity of HCC cells through the transport of relevant proteins via EVs. Studies have shown that genetically engineered HCC cells expressing the sodium iodide symporter (NIS) protein can transfer NIS protein to other HCC cells in the form of EVs, exhibiting a high sensitivity to radiotherapy [136].

There are also several proteins with drug potential that deserve attention. The TRAIL protein, functioning as a transmembrane death receptor ligand, engages with cell surface receptors DR4 and DR5, prompting cellular apoptosis through Caspase-8-mediated signaling cascades. Evidence suggests that EV-delivered TRAIL effectively induces apoptosis in various cancer cell lines, including lung cancer and breast cancer. Moreover, several studies have identified a synergistic effect between TRAIL and sorafenib. This suggests that EV-mediated delivery of TRAIL may offer a promising therapeutic strategy to overcome drug resistance in HCC [18]. One recent study indicated that the Parkinson’s disease-related protein α-synuclein can be secreted in the form of EVs and can traverse the blood-brain barrier. It has been found to inhibit the proliferation and migration of HCC [137].

Based on the current research progress, it can be seen that studies on the application of EV-related proteins in HCC treatment are mostly at the cellular experiment stage. Some therapeutic potentials were confirmed in animal models; however, there is still a long way to go before clinical trials in humans can be conducted, such as the lack of standardized technologies for large-scale EVs production. In Table 2, we summarized the current research progress about the treatment of EV-associated proteins.

**Table 2:** The current progress of EV-associated proteins for the treatment of HCC

Protein	Origins	Location	Effects	Ref.
LAMP2B	Naturally present in various types of EVs	Membrane	Serve as a kind of protein anchor	[128]
SP94-LAPM2B-RRM complex	Transfection from plasmids into HEK-293T cells	Membrane	Enhance sorafenib-induced ferroptosis in HCC by silencing GPX4 and DHODH expression	[129]
CD47	Transfection from plasmids into HEK-293T cells	Membrane	Ensure the EVs effectively escape the phagocytosis of mononuclear phagocyte system/ Induces ferroptosis in HCC with reduced toxicity when combined with Rose Bengal (a kind of photosensitizer) and Erastin (a kind of ferroptosis inducer)	[130]
Cas9 ribonucleoprotein	A kind of protein complexed with sgRNA which can be loaded into EVs isolated from LX-2 cells through electroporation	Cargo	Enable the efficient delivery of CRISPR-Cas9 system/ Cas9 RNP targeting lysine acetyltransferase 5 (*KAT5*) can suppress the growth of HCC	[131]
CP05	Naturally present in various types of EVs	Membrane	Serve as a kind of protein anchor	[16, 132]
N1ND-CP05 complex	Incubated with EVs from Hepa1-6 cells	Membrane	Activate dendritic cells and activate memory T cells	[132]
N1ND-CP05& AFP212- CP05& P47-CP05 complex	Incubated with EVs from DC2.4 cells	Membrane	Promote recruitment, accumulation and activation of dendritic cells/ Enhance cross-presentation of tumor neoantigens and T cell response	[16]
MS-275	A kind of histone deacetylase inhibitor	NA	Increase the expression of HSP70 and MICB in EVs from HepG2 cells and augment the cytotoxicity of NK cells	[133]
5-Aza-2’-deoxycytidine	A kind of DNA methyltransferase	NA	Increase the expression of HLA-I and NY-ESO-1 in EVs from HepG2 and Hep3B cells and stimulate anti-tumor-specific immune response	[135]
HSP60&HSP70&HSP90	Naturally present in tumor-derived EVs	Cargo	Stimulate NK cell cytotoxicity and induce HSP-specific NK cell anti-tumor responses	[17]
NIS	Transfection from plasmids into Huh7 cells	Cargo	Increase the I^131^ radioiodine uptake and DNA damage of HCC cells, show the potential to revert radioiodine-resistant cancers into radioiodine-sensitive cancers	[136]
TRAIL	A kind of ligand to the death receptors, DR4 and DR5, which can be encapsulated into EVs	Cargo	Induce cellular apoptosis of tumor cells through the Caspase-8-mediated signaling cascades	[18]
α-synuclein	A main component of of Lewy bodies secreted by SH-SY5Y cells/ Transfection from plasmids into HEK-293T cells	Cargo	Inhibited the cell viability, migration, and invasion of HCC cells	[137]

Ref., reference; NA, not available

## Conclusions and future prospects

This review introduces the biological characteristics and biogenesis of EVs, emphasizes the role of EV-associated proteins in the development of HCC, and summarizes current research on EV-associated proteins as tumor biomarkers and therapeutic targets for HCC. EVs, as important mediators of intercellular communication, play a significant role in the tumor progression of HCC. We have discussed the functions of EV-associated proteins from the perspectives of communication between HCC and components of the TME, and mechanisms of tumor metastasis including EMT and targeted metastasis. We outlined the advantages of EV-associated proteins as biomarkers for HCC and provided diagnostic data from existing studies, demonstrating their excellent diagnostic efficacy. From the perspective of therapeutic exploration, we have summarized that EV-associated proteins can serve as anchors for drug delivery, improving the accuracy of drug delivery. Additionally, they can directly exhibit anti-tumor effects, demonstrating promising therapeutic potential.

By reviewing existing research, we believe that the challenges identified in current studies can provide valuable insights for future research directions. Firstly, the protein composition of EVs is diverse and complex. In the same tumor process, EVs may contain proteins with opposite functions. For clinical applications, it is necessary to accurately isolate proteins in EVs that inhibit tumors and further consider methods for their effective delivery. Secondly, EV-associated proteins as tumor biomarkers for HCC hold significant clinical potential due to their stability and resistance to degradation. However, the high cost of EV extraction and the lack of standardized biomarkers to determine EV origin are challenges. Currently, most relevant research has not yet entered the clinical stage, and many potential protein markers lack diagnostic test data, highlighting the urgent need for further clinical validation in large cohorts. Thirdly, the exploration of therapeutic potential associated with EV-associated proteins warrants further investigation. In contrast to the extensive exploration of systemic treatment strategies for advanced HCC, such as targeted and immunotherapy combinations, there is limited research exploring the intersection of EV-associated proteins with current systemic treatment studies for advanced HCC.

Despite the challenges faced by current research, the undeniable potential of EV-associated proteins in the clinical application of HCC is evident. In conclusion, research on EV-associated proteins holds the promise to deepen our understanding of HCC and provide a unique perspective for the development of new treatment strategies.

## Data Availability

Not applicable.
